# Supplementary material for: Identification of photoperiod-induced specific miRNAs in the adrenal glands of Sunite sheep (Ovis aries)
Source: Front Vet Sci. 2022 Jul 22;9:888207. doi: 10.3389/fvets.2022.888207 (PMC9354845; doi:10.3389/fvets.2022.888207)
Supplement: Supplementary file 1 [file Data_Sheet_1.ZIP › Supplementary file/miRDeep2 file.zip!/miRDeep2 file/Know_Structure/LP42A3/oar-mir-3955.pdf]

```
miRBase precursor            : oar-mir-3955
Total read count             : 1203
oar-miR-3955-5p read count   : 1070
oar-miR-3955-3p read count   : 93
remaining reads              : 40
```

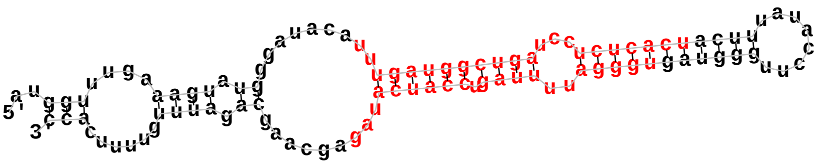

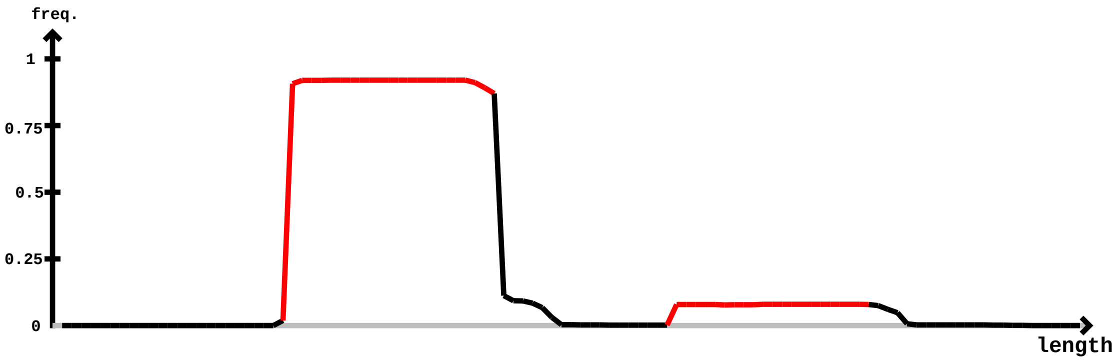


```
                          oar-miR-3955-5p
                                                        oar-miR-3955-3p
5'- augguuugaaaguaugggauacauuugauggcugauccucucacuacuuuauaccuuggguagugggauuuuaguccaucauagagcaagcagauuuguuuucacc   -3'   exp
    ...........((((...))))...((((((((((...(((((((((((........)))))))))))..)))).)))))).(((((((......)))))))....   reads   mm   sample
    ......................auuugauggcugauccucuc................................................................   3       0    seq
    ......................auuugauggcugauccucucac..............................................................   1       0    seq
    ......................auuugauggcugauccucucacu.............................................................   15      0    seq
    ......................auuugauggcugauccucucacuacuu.........................................................   1       0    seq
    ......................auuugauggcugauccucucacuacuuu........................................................   2       0    seq
    ......................auuugauggcugauccucucacuacuuua.......................................................   1       0    seq
    .......................uuugauggcugauccucuc................................................................   8       0    seq
    .......................uuugauggcugauccucuca...............................................................   23      0    seq
    .......................uuugauggcugauccucucac..............................................................   25      0    seq
    .......................uuugauggcugauccucucacu.............................................................   888     0    seq
    .......................uuugauggcugauccucucacua............................................................   22      0    seq
    .......................uuugauggcugauccucucacuac...........................................................   1       0    seq
    .......................uuugauggcugauccucucacuacu..........................................................   8       0    seq
    .......................uuugauggcugauccucucacuacuu.........................................................   16      0    seq
    .......................uuugauggcugauccucucacuacuuu........................................................   41      0    seq
    .......................uuugauggcugauccucucacuacuuua.......................................................   32      0    seq
    .......................uuugauggcugauccucucacuacuuuaua.....................................................   1       0    seq
    .......................uuugauggcugauccucucacuacuuuauaccu..................................................   1       0    seq
    .......................uuugauggcugauccucucacuacuuuauaccuuggguaguggga......................................   2       0    seq
    ........................uugauggcugauccucucacu.............................................................   9       0    seq
    ........................uugauggcugauccucucacua............................................................   1       0    seq
    ........................uugauggcugauccucucacuacu..........................................................   2       0    seq
    ........................uugauggcugauccucucacuacuu.........................................................   2       0    seq
    ........................uugauggcugauccucucacuacuuu........................................................   1       0    seq
    ...........................auggcugauccucucacuacu..........................................................   1       0    seq
    ...............................................................ugggauuuuaguccaucaua.......................   1       0    seq
    ...............................................................ugggauuuuaguccaucauag......................   5       0    seq
    ...............................................................ugggauuuuaguccaucauaga.....................   17      0    seq
    ...............................................................ugggauuuuaguccaucauagag....................   15      0    seq
    ...............................................................ugggauuuuaguccaucauagagc...................   50      0    seq
    ...............................................................ugggauuuuaguccaucauagagca..................   5       0    seq
    .....................................................................uuuaguccaucauagagcaagcagauu..........   1       0    seq
    ........................................................................aguccaucauagagcaagcagauuug........   1       0    seq
    ........................................................................aguccaucauagagcaagcagauuuguu......   1       0    seq
```

oar-miR-3955-5p

oar-miR-3955-3p

augguuugaaaguaugggauacauuugauggcugauccucucacuacuuuauaccuuggguagugggauuuuaguccaucauagagcaagcagauuuguuuucacc
